# Association Between Caregivers’ and Care-Recipients’ Advance Care Planning: An Exploratory Study

**DOI:** 10.1093/geroni/igaa057.1343

**Published:** 2020-12-16

**Authors:** Hyunjin Noh, Lewis Lee, Chorong Won

**Affiliations:** The University of Alabama, Tuscaloosa, Alabama, United States

## Abstract

Research on advance care planning (ACP) has highlighted major contributors to the completion of ACP documents. One of such contributors is knowledge about ACP, such as an advance directive or living will (LW). This study aims to 1) understand the initial exposure to ACP knowledge among informal caregivers’ of chronically or seriously ill older adults and to 2) explore an association between caregivers’ advance care planning and that of their care-recipients. Forty-four primary caregivers of cognitively impaired older adults were recruited at various community settings. A mixed-method design was used to qualitatively interview each participant face-to-face about his or her initial experience with ACP and to quantitatively ask if the participant completed a LW and if the care-recipient completed one as well. Qualitative content analysis of participant responses revealed that their initial experiences with ACP were mostly through their care-recipients, such as the care-recipient’s ACP in previous hospitalizations or legal consultations. Chi-square test for independence was conducted to explore whether there is an association between caregivers’ LW completion and that of care-recipients. The results show that there is a significant relationship between the two variables: χ2 (1, n = 44) = 8.84, p < .001, φ = .49. These findings suggest that secondary experiences with close one’s ACP may serve as facilitator to one’s ACP completion. Therefore, efforts to promote ACP should target a caregiver and care-recipient dyad so that caregivers as well as care-recipients may learn about and complete ACP documents.

